# A Novel Technique of Hemorrhage Control for an Inferior Vena Cava Injury From an Abdominal Stab Wound Using Two Balloon Occlusion Catheters: A Case Report

**DOI:** 10.7759/cureus.81009

**Published:** 2025-03-22

**Authors:** Yuki Mochida, Koya Sekiguchi, Hirotaka Nishimura, Yasuhiko Kaita, Yoshihiro Yamaguchi

**Affiliations:** 1 Department of Trauma and Critical Care Medicine, Kyorin University School of Medicine, Tokyo, JPN

**Keywords:** balloon catheter, emergency vascular injuries, resuscitative endovascular balloon occlusion of the aorta, trauma-related vascular injury, vascular repair

## Abstract

Penetrating injuries to the inferior vena cava (IVC) caused by stab wounds are uncommon; however, mortality risk is high due to substantial bleeding. Managing these injuries is particularly challenging because of their retroperitoneal location and high venous flow, which complicate surgical interventions. Conventional treatments, including direct suturing and graft placement, are often difficult to perform in unstable patients. Although endovascular approaches are frequently used for aortic injuries, there is limited data for managing IVC injuries. Here, we present a novel case in which hemostasis was achieved using two intravascular balloon catheters, offering insights into a potentially effective technique for life-threatening vascular trauma. We report a case of a man in his 50s who was admitted to our hospital after a self-inflicted stab wound to the upper abdomen. At presentation, the patient was hemodynamically stable; however, imaging revealed a hematoma around the IVC below both renal veins, suggesting vessel injury. Bleeding was initially controlled with two balloon catheters positioned proximally and distally to the site of the suspected injury. The balloons were inflated to achieve temporary hemostasis, followed by surgical exploration. A 2.5 cm longitudinal tear on the right lateral wall of the IVC was identified and repaired using continuous sutures. Postoperatively, the patient recovered uneventfully, although a pulmonary embolism (a branch of the right pulmonary artery) without respiratory compromise was identified on imaging. Anticoagulant therapy was initiated, and no further complications were observed. This case demonstrates a novel option for hemorrhage control in IVC injuries by deploying two intravascular balloon catheters. This technique allows for the effective management of unexpected intraoperative bleeding and minimizes blood loss, suggesting its potential application in the treatment of complex vascular trauma. Further studies are needed to refine the indications for balloon deployment, placement techniques, and thrombosis prevention strategies to improve patient outcomes.

## Introduction

Inferior vena cava (IVC) injuries caused by penetrating trauma, such as stab wounds, are rare but life-threatening conditions with high mortality rates [1-2]. Owing to the retroperitoneal location, proximity to vital organs, and substantial blood flow, managing IVC injuries presents unique challenges [1]. Uncontrolled bleeding can rapidly lead to hypovolemic shock and death, making hemorrhage control critical for successful treatment [2]. Traditional management involves open surgical repair including direct suturing, patch angioplasty, or graft placement, which can be technically demanding in unstable patients [3]. Recently, endovascular techniques such as resuscitative balloon occlusion of the aorta (REBOA) have emerged as adjunctive tools for temporary blood flow control and improved outcomes in patients [4-6]. While REBOA is widely used for temporary hemorrhage control in trauma patients, its application in IVC injuries has been less explored. We report a case of IVC injury caused by an abdominal stab wound in which hemorrhage control was attempted using two intravascular balloon catheters, similar to REBOA. This case demonstrates the potential utility of endovascular methods for controlling massive bleeding in IVC injuries, particularly in situations where traditional surgical techniques may pose significant challenges.

## Case presentation

**Figure 1:**
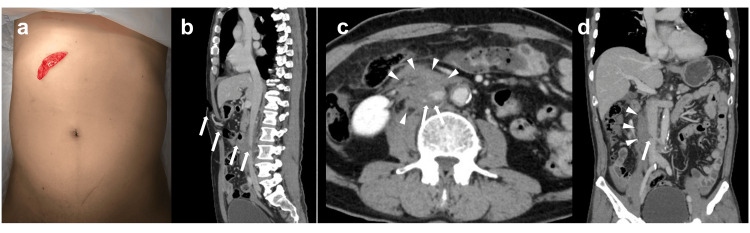
Photographs of the wound and computed tomography (CT) findings at the time of admission. a: Upon reducing the exposed mesentery into the abdominal cavity, a 5 cm stab wound was observed slightly below the right costal margin. b: Contrast-enhanced sagittal CT shows the stab trajectory extending slightly caudally from the body surface to the inferior vena cava (IVC) (arrow). c: Axial contrast-enhanced CT reveals a contrast defect in the IVC below the renal veins (arrow) and a surrounding hematoma (arrowhead). d: Coronal contrast-enhanced CT demonstrates a contrast defect on the right side of the IVC (arrow) with a hematoma extending from this point (arrowhead).

A 50-year-old male with no significant medical history (body mass index: 22.8) was brought to our emergency center by an ambulance approximately 40 min after attempting suicide by stabbing himself in the upper abdomen using a kitchen knife. On arrival, the patient was alert and conscious, with a pulse of 82 bpm, blood pressure of 114/60 mmHg, oxygen saturation of 100% on room air, and body temperature of 36.6℃. No significant abnormalities were observed in the blood test results. A stab wound approximately 5 cm in length was observed just below the right costal margin, with the mesentery protruding through the wound (Fig. 1a).

Ultrasonography did not reveal any specific findings, such as ascites accumulation. Contrast-enhanced abdominal computed tomography (CECT) performed approximately 15 minutes after arrival revealed that the knife had penetrated obliquely in the caudal direction (Fig. 1b). Although there was no active extravasation on CECT, a hematoma was noted inferior to both renal veins around the IVC (Fig. 1c). The IVC had a contrast defect approximately 4 cm below the renal vein level, which was assumed to be the injury site (Fig. 1d). Based on these findings, we diagnosed the patient with an IVC injury and decided to perform emergency laparotomy. As the patient's overall condition was stable and there was time to prepare the operating room, it was decided to place an intravascular balloon catheter in the IVC to prevent massive bleeding during surgical repair. After intubation, the patient was transferred to the angiography room approximately one hour after arrival.

**Figure 2:**
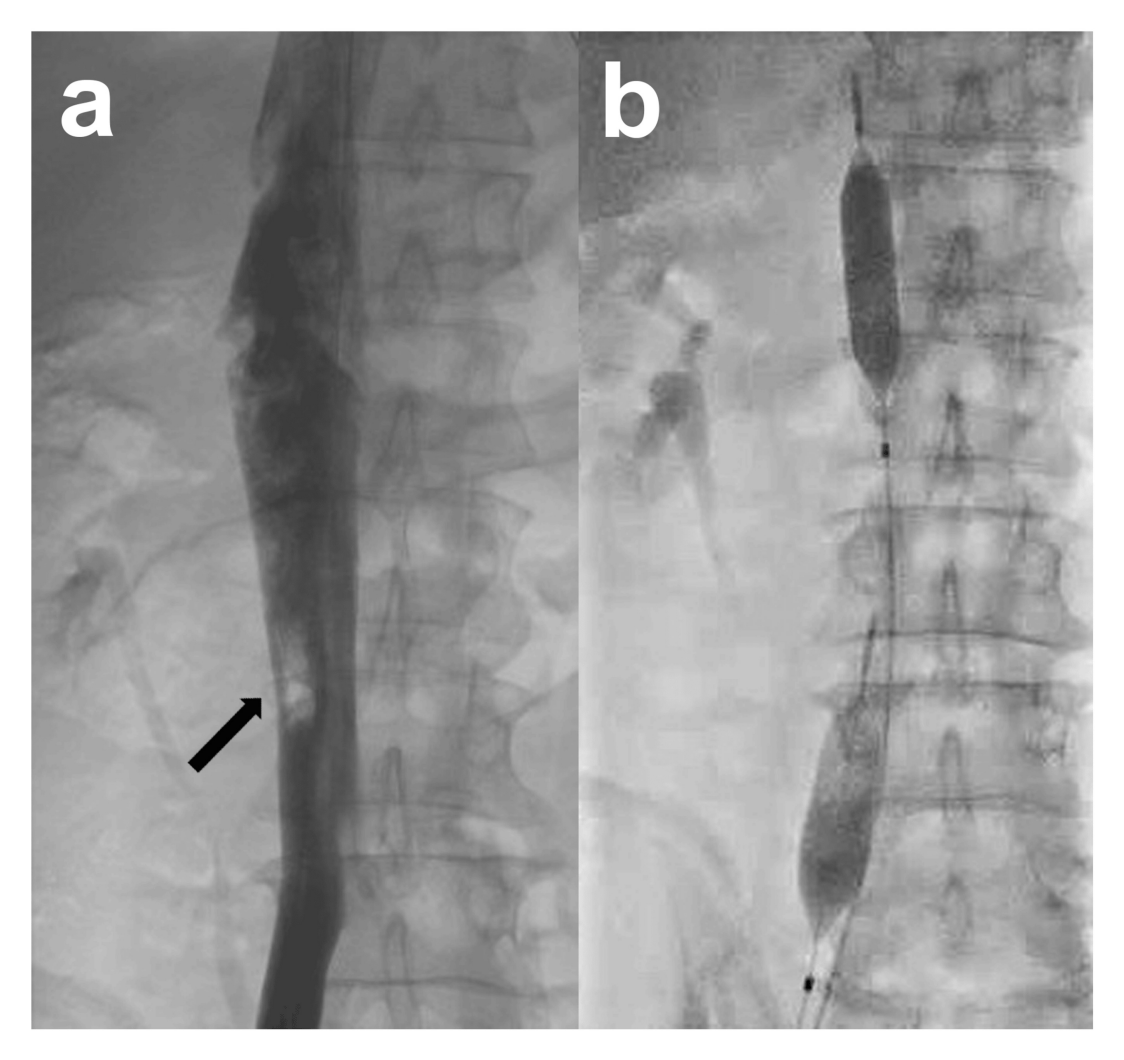
Angiography findings. a: Digital subtraction angiography of the inferior vena cava (IVC) performed through a sheath inserted into the right femoral vein shows a contrast defect at the site of injury (arrow). b: A fluoroscopic image showing the insertion of two balloons and their inflation to a volume sufficient for complete occlusion. The distal balloon was located between the right common iliac vein and the IVC.

Two rescue balloon catheters (Rescue Balloon-ER; Tokai Medical Products, Aichi, Japan) were inserted into the right femoral vein using a 7Fr vascular sheath. Under fluoroscopic guidance, angiography confirmed the location of the IVC injury, and the balloons were positioned proximally and distally to the injury site (Fig. 2a, 2b). Under fluoroscopic guidance, the required inflation volume was determined using a contrast agent to achieve complete balloon occlusion. During the procedure, hemodynamics remained stable. Subsequently, the balloons were deflated and the patient was transferred to the operating room. The angiographic procedure, including the time required for transfer to the angiography room, took 70 minutes.

**Figure 3:**
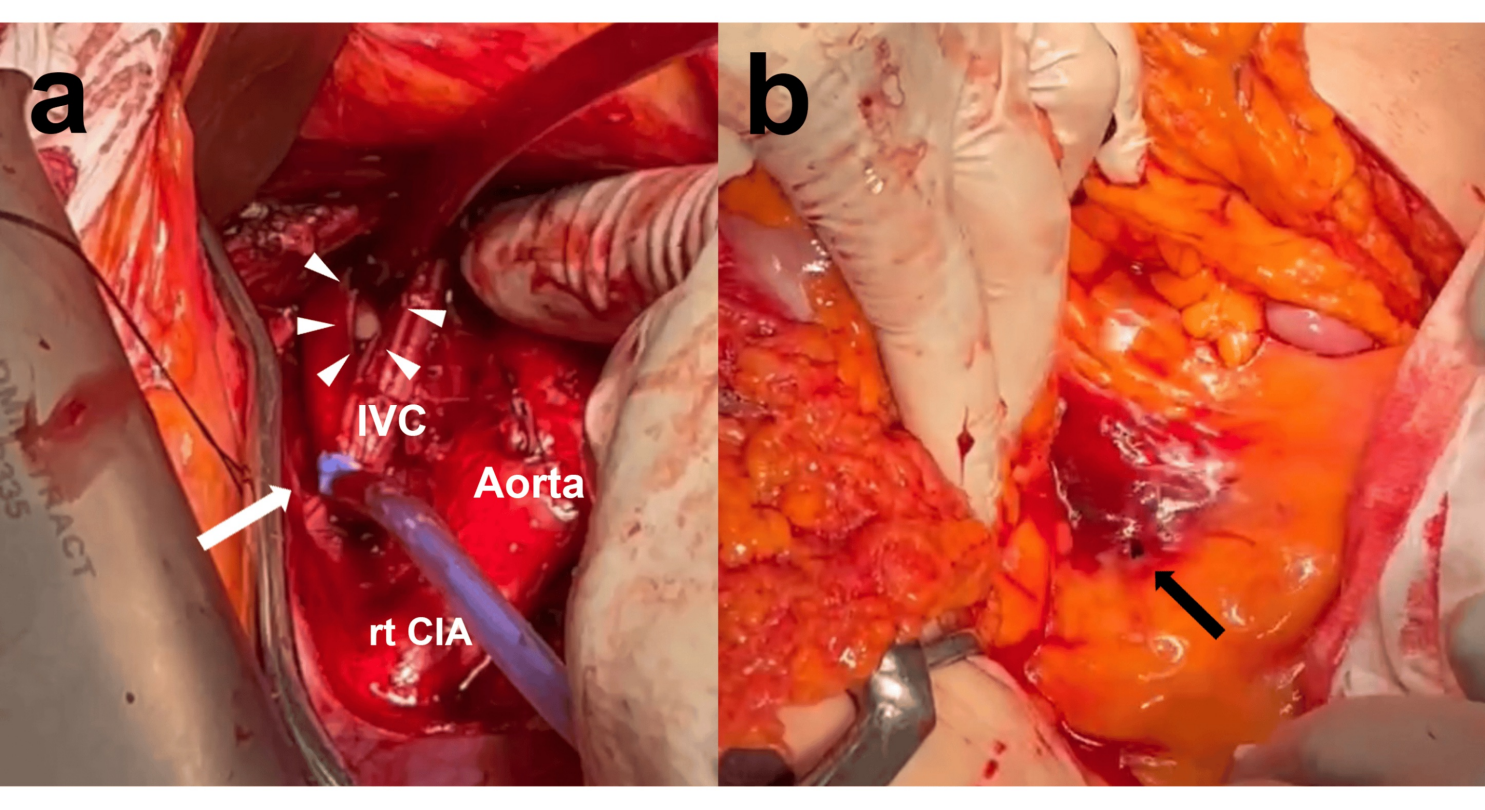
Intraoperative findings. a: The proximal side of the IVC was occluded with a balloon, and the distal side was snared using a vascular tape tourniquet (arrow), allowing effective bleeding control and visualization of the injury site (arrowhead). b: The injury to the mesentery leading to the IVC damage was identified as a 5 mm stab wound (arrow).

A midline abdominal incision was made, and hemoperitoneum was observed upon entering the peritoneal cavity. To secure access to the retroperitoneum, the mesentery was retracted into the upper right abdominal cavity. The abdominal aorta was identified at the position of the mesentericoparietal recess, and careful dissection was performed to separate the hematoma-containing adipose tissue on its right side, allowing identification of the IVC. An accessory branch of the right renal artery (toward the lower pole) coursing directly above the IVC was encountered and subsequently ligated to improve access. Upon dissecting the surrounding tissues and exposing the IVC, venous bleeding was observed, and a large defect was identified on the right lateral wall of the IVC, which was palpated and confirmed with a finger. Immediate digital compression of the defect was performed to control bleeding, which was effective but obstructed the surgical field. Therefore, the previously placed occlusion balloons were inflated to the complete IVC occlusion both proximally and distally. Although significant hemostasis was achieved to the extent that the defect in the injury site was visible, a small amount of bleeding persisted. This was presumed to be due to uncontrolled retrograde flow from the lumbar veins and inflow from the left common iliac vein. Therefore, to further reduce blood loss and ensure a clear surgical field, it was decided to control blood flow distal to the injury site using a tourniquet instead of the distal balloon. While maintaining digital compression over the defect, the area around the IVC immediately above the confluence of the right and left common iliac veins was dissected. Vascular tape was then placed around the IVC and secured with a tourniquet snare just proximal to the distal balloon to achieve better hemostasis. With proximal balloon occlusion and distal tourniquet control successfully achieving hemostasis, the IVC injury could be fully inspected. A longitudinal laceration approximately 2.5 cm in length was identified (Fig. 3a). Although backflow from the lumbar veins was noted, it was managed with suction and the defect was repaired using continuous suturing with 5-0 polypropylene sutures. Before completely closing the laceration, the vascular lumen was filled with blood to ensure that no air remained in the vessel. Subsequently, the repair was completed, the proximal balloon was deflated, and a distal tourniquet snare was released. The balloon inflation time during surgery totaled 16 minutes. Hemostasis was confirmed at the suture site of the IVC, and the retroperitoneal space was closed. Further inspection revealed a stab wound in the mesentery that appeared to have reached the retroperitoneal IVC. The mesenteric wound was smaller than the IVC injury, with an approximately 5 mm defect (Fig. 3b). No other injuries were observed and the abdomen was closed. 

**Figure 4:**
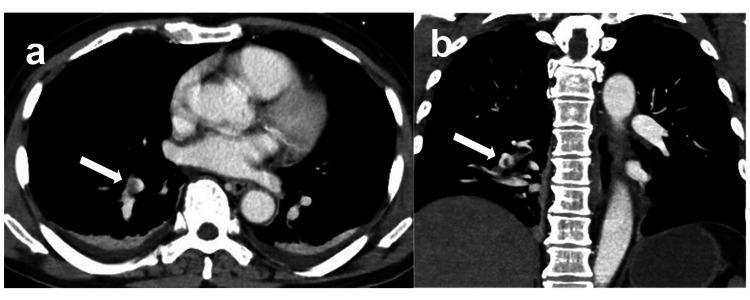
Postoperative contrast-enhanced CT findings of the torso. a: Contrast-enhanced axial CT shows a thrombus in a branch of the right pulmonary artery (arrow). b: Contrast-enhanced coronal CT shows a thrombus in a branch of the right pulmonary artery (arrow).

During the surgery, four units of packed red blood cells were transfused. Hemoglobin levels were 13.2 g/dL preoperatively and 11.3 g/dL postoperatively. Both balloon catheters were removed immediately after surgery, and the vascular sheath was removed on the following day. Postoperatively, the patient showed no progression of the anemia and had an uneventful recovery. The patient was weaned from mechanical ventilation and extubated on the first postoperative day. On the third postoperative day, CECT performed for follow-up incidentally revealed a pulmonary embolism (PE) in a branch of the right pulmonary artery (Fig. 4). No thrombus was observed at the repaired IVC site nor at the sheath insertion site in the right femoral vein. As the patient was hemodynamically stable and exhibited no respiratory symptoms, anticoagulant therapy for low risk with edoxaban (60 mg/day) was initiated. The patient experienced a prolonged rehabilitation period due to the PE but had a favorable postoperative course and was discharged on postoperative day 11.

## Discussion

IVC injuries are associated with unique risks due to the vessel's high-flow dynamics and anatomical challenges. The retroperitoneal location often results in a delayed diagnosis, as bleeding may initially be contained within the retroperitoneal space. Once diagnosed, rapid intervention is required to prevent catastrophic hemorrhage and ensure patient survival [1-3]. In this case, the trajectory of the knife within the body was assessed using CECT, and the presence of a hematoma around the IVC made the suspicion of IVC injury relatively straightforward. 

Traditional open repair methods require extensive surgical exposure, which not only prolongs the operative time but also increases the risk of intraoperative mortality, particularly in unstable patients [1-2]. REBOA is a well-known technique for temporarily controlling hemorrhage by placing a balloon in the aorta. Its application to the vena cava has been reported in animal studies and is referred to as REBOVC (resuscitative balloon occlusion of the inferior vena cava) [7-8]. These previous reports have demonstrated the feasibility of balloon placement in the IVC through animal studies; however, specific strategies regarding the placement location and method in clinical settings have yet to be established. Initially, we deployed a single balloon at the injury site to occlude the defect. However, two potential issues were identified: (1) maintaining the balloon in an inflated state during repair could obstruct the surgical field and hinder suturing, and (2) inflation of the balloon at the injury site could further enlarge the defect. Therefore, we opted to achieve hemorrhage control by placing balloons both proximally and distally to the site of injury for occlusion. Although a previous animal study has reported the use of double-balloon techniques for IVC hemorrhage control, no commercially available double-balloon products are suitable for clinical application [9]. Consequently, to expedite hemorrhage control, we inserted two sheaths into the right femoral vein and deployed two rescue balloon catheters. 

In the present case, the injury site was located near the confluence of the bilateral common iliac veins. While we attempted to position the distal balloon to occlude the inflow from the left common iliac vein as much as possible, complete control of the blood flow from the left common iliac vein was not achieved. Consequently, we decided to directly occlude the distal IVC within the surgical field for more effective hemorrhage control. The IVC near the confluence of the bilateral common iliac veins provides relatively good exposure, making taping of the IVC straightforward. As balloon catheters had already been inserted, we opted to use a tourniquet for blood flow occlusion, rather than vascular clamps. Owing to balloon occlusion, the patient's vital signs remained stable throughout the surgery with a minimal requirement for red blood cell transfusions.

The proximal balloon was effective in achieving hemostasis, suggesting that dual-balloon hemorrhage control may be particularly beneficial for high-risk IVC injuries located posterior to the liver, pancreas, or duodenum [10]. In such cases, the proximal and distal balloons can be positioned at appropriate distances, enhancing their utility in managing challenging bleeding scenarios. 

Endovascular tools such as balloon catheters are increasingly being recognized as valuable for managing complex vascular trauma [11]. In addition to temporary hemorrhage control, they minimize blood loss and may reduce the need for more invasive maneuvers such as full vascular isolation or proximal ligation [12]. Even in cases of IVC injury below the renal vein confluence, as in this case, we hypothesize that the use of balloon occlusion offers the advantage of reducing the extent of proximal dissection and surgical exposure compared to conventional techniques. Traditionally, surgical management of IVC injuries requires performing the Cattell-Braasch procedure, which is an established technique for accessing retroperitoneal organs [13-14]. This is followed by applying pressure to both the distal and proximal parts of the IVC with a sponge stick to identify the injury site. While this approach provides effective exposure, it may require the surgeon's skill in certain cases and does not always guarantee reliable hemorrhage control. In contrast, balloon occlusion can shorten the procedure time and reduce blood loss, improving overall surgical efficiency and safety. As the IVC is located in the retroperitoneal space, bleeding may be contained within the retroperitoneum, which can result in hemodynamic stability upon arrival, as observed in this case. However, once the retroperitoneum is opened during surgery, there is a risk of massive bleeding from the injury site. Ideally, the procedure should proceed smoothly, allowing for prompt identification of the injury site and effective blood flow control with vascular clamps. However, in cases where locating the injury is challenging or hemorrhage becomes uncontrollable, the preemptive placement of a balloon catheter serves as a valuable backup strategy. This approach can enhance surgical safety and facilitate effective hemorrhage management.

On the other hand, balloon catheter application requires careful planning as improper deployment or prolonged use can lead to complications, including thrombosis or distal ischemia. Moreover, balloon occlusion can cause blood stasis, and complications such as PE and venous thromboembolism have been reported following the use of REBOA [12,15-16]. In this case, the balloons were placed in the IVC, with a potentially equal or greater risk of thrombosis than aortic balloon occlusion. Nonetheless, the PE observed during the patient’s postoperative course was a significant complication. Although effective strategies for thrombosis prevention are undefined, in an effort to reduce the thrombosis risk in this case, the balloon inflation time was minimized to only the duration required for distal tourniquet snaring and IVC suturing. Despite these precautions, thrombosis occurred, possibly due to the use of two balloons or the use of the same site for vascular sheath insertion. In this case, balloon insertion from the jugular vein toward the IVC was avoided due to concerns about potential injury to the right atrium or thromboembolism into the right atrium. To minimize procedure time, the balloon was inserted via the ipsilateral femoral vein. However, the optimal insertion route for the balloon remains a subject for further investigation.

Systemic heparinization carries risks in cases of multiple trauma; however, in this case, where the bleeding source was presumed to be limited to a penetrating injury of the IVC, prophylactic administration of heparin before balloon inflation might have been beneficial.　This indicates the need to consider thrombosis risk-mitigation strategies tailored to each case. However, given that the precise risk of thrombosis remains unclear, it may be advisable to avoid the routine use of balloon occlusion. Instead, it should be considered as an adjunctive measure, reserved for intraoperative emergencies when necessary.

Additionally, the potential for additional time required for balloon insertion should be considered. In this case, there was sufficient time for pre-operative insertion, and the procedure was performed to reduce surgical risk. However, its effectiveness in cases where bleeding persists after arrival and hemodynamic stability cannot be maintained remains unknown. Moreover, in such cases, the injury site may be larger, potentially increasing the risk of guidewire misplacement and other complications. Considering these factors, balloon occlusion for IVC injuries should be applied in cases where hemodynamic stability allows for sufficient time, with the primary aim of reducing intraoperative bleeding risks. It is preferable to use balloon occlusion as an adjunctive measure rather than a primary intervention.

## Conclusions

IVC occlusion using two balloon catheters may be an effective method for reducing intraoperative blood loss and minimizing the need for transfusion in life-threatening IVC injuries, particularly in cases of retrohepatic IVC injury. While this technique is not intended to completely replace conventional surgical procedures such as the Cattell-Braasch maneuver, it may serve as a valuable adjunctive option. With the accumulation of similar cases in the future, it may become possible to evaluate and compare factors such as balloon placement duration, associated hemodynamic changes, risk of thrombosis, transfusion requirements, and length of hospital stay between cases managed with and without this technique. Such findings could contribute to establishing this method as a standardized approach.
